# Moderate Altitude Affects High Intensity Running Performance in a Collegiate Women’s Soccer Game

**DOI:** 10.1515/hukin-2015-0070

**Published:** 2015-10-14

**Authors:** Jonathan D. Bohner, Jay R. Hoffman, William. P. McCormack, Tyler C. Scanlon, Jeremy R. Townsend, Jeffrey R. Stout, Maren S. Fragala, David H. Fukuda

**Affiliations:** 1 Institute of Exercise Physiology and Wellness, University of Central Florida, Orlando, USA.

**Keywords:** athletes, sport science, sprinting, hypoxia, competition

## Abstract

The effect of altitude on soccer game activity profiles was retrospectively examined in six NCAA Division I female soccer players. Comparisons were made between two matches played at sea level (SL) and one match played at a moderate altitude (1839 m). A 10-Hz global positioning system device was used to measure distance and velocity. The rate of total distance capacity (TDC) and high intensity running (HIR) as well as percent of time at HIR were evaluated. Significant differences were seen in the distance rate (120.55 ± 8.26 m·min−1 versus 105.77 ± 10.19 m·min−1) and the HIR rate (27.65 ± 9.25 m·min−1 versus 25.07 ± 7.66 m·min−1) between SL and altitude, respectively. The percent of time at HIR was not significantly different (p = 0.064), yet tended to be greater at SL (10.4 ± 3.3%) than at altitude (9.1 ± 2.2%). Results indicate that teams residing at SL and competing at a moderate altitude may have a reduced ability in distance covered and a high intensity run rate.

## Introduction

The game of soccer incorporates both aerobic and anaerobic components which need to be sustained throughout a match (Billaut et al., 2012; Gore et al., 2008; Krustrup et al., 2005; Mohr et al., 2003). In general, each soccer game consists primarily of prolonged sub-maximal activity, interspersed with explosive bouts of sprinting (Bradley et al., 2009; Krustrup et al., 2005; Mohr et al., 2003). This change in pace and exertion, as well as a player’s ability to recover from repetitive sprints are critical to game performance.

The acute physiological changes associated with varying altitudes above sea level may have significant effects on performance, especially in athletes that are not acclimatized (Wehrlin and Hallen, 2006). Acute performance at altitude can impair aerobic performance and increase the rate of fatigue for athletes who ascend from sea level. A simulated change in altitude from 300 m to 2,800 m has been shown to result in a significant decrease in oxygen saturation, time to exhaustion and VO2max during exercise (Wehrlin and Hallen, 2006). These investigators reported a 6.3% decrease in VO2max per 1,000 m of increasing altitude. As individuals ascend to altitude, a decrease in stroke volume is seen resulting from a reduced cardiac preload due to hemoconcentration causing a compensatory increase in the heart rate (Buchheit et al., 2012; Grover et al., 1986). Depending upon the altitude, these cardiovascular changes can cause significant performance decrements in activities that are primarily dependent upon aerobic metabolism. However, the effects on high intensity activity may be different. Evidence suggests that sprint performance may actually be enhanced due to the lower air resistance, resulting in less drag force (Arsacm, 2002; Levine et al., 2008; Peronnet et al., 1991). However, the ability to recover from multiple sprints may be affected by impaired cardiovascular function associated with altitude. This has potential implications for competitive sporting events played at altitude.

Living at altitude results in specific physiological adaptations (e.g., an increase in red blood cell production, hemoglobin concentration, blood volume and buffering capacity) that enhances cardiovascular performance, and may provide an advantage for athletes residing at altitude (Levine and Stray-Gundersen, 1997). For sports such as soccer, matches at altitude may present a distinct disadvantage for teams that reside at sea level (Bartsch et al., 2008; McSharry, 2007). Due to the concern for a potential performance advantage for teams residing at altitude, the Fédération Internationale de Football Association (FIFA) had considered a ban on international matches at elevations above 2500 m (McSharry, 2007).

The total distance covered in a soccer game has been reported to be decreased up to 3% at altitudes above 1200 m (Nassis, 2013). However, studies examining the effects of an acute match at a moderate altitude are limited. Some investigations have observed high intensity running and analyzed game performance using videotape to quantify movement during a match (Bradley et al., 2009; Krustrup et al., 2005; Mohr et al., 2003). The outcomes of these studies suggest that the exertion rate and frequency of high intensity running among elite soccer players are altered during a match with an earlier onset of fatigue. However, technological advances in analyzing player activity variables/profiles though the use of global positioning systems (GPS) provides a more sensitive measure for monitoring performance in multiple players during actual matches. Thus, the purpose of this investigation was to compare the effect of a moderate altitude on the rate of high intensity running and distance run during competitive games in women’s intercollegiate soccer.

## Material and Methods

### Experimental Design

Three Division I women’s soccer matches were retrospectively analyzed to assess high intensity running performance throughout the duration of a competitive soccer game. Two matches performed at sea level (Orlando, FL; 25 m) were averaged to establish a baseline performance measure, followed by a match in Colorado Springs, Colorado (1839 m), which was considered to be the game performed at a moderate altitude. The team arrived in Colorado Springs the day before the game. Games analyzed were performed during three consecutive weeks. Each match was played on a Friday evening and was separated by five days from the previous match (the team also played on each Sunday afternoon). All matches were played towards the end of the regular season, during the month of October. Thus, it was assumed that the conditioning level of the athletes would be high and remain similar within that three week time span. All games played were classified as close games (outcome of each contest was within 2 goals), suggesting that strategy and substitution patterns of the coach would be similar in all contests. Differences in the environmental conditions between games are depicted in Table 1. Consideration for player analysis was determined by time played during each match. A minimum of sixty minutes played per game was used as the threshold to consider the player for inclusion in the analysis. Following this exclusion factor, data from six de-identified players were used in this retrospective analysis. During all matches players wore global positioning devices (GPS). Comparisons of the rate of high intensity running (HIR) and total distance covered (TDC) were made between games played at sea level and at altitude.

### Participants

Retrospective examination of six National Collegiate Athletic Association (NCAA) Division I women soccer players (19.5 ± 1.0 y; 165.2 ± 5.5 cm; 62.1 ± 6.4 kg, 50.1 ± 3.3 ml·kg·min^−1^) was performed. The athletes’ playing positions consisted of defenders (2), midfielders (2), and forwards (2). All performance assessments were part of the athletes’ regular in-season assessment protocol that had been designed to provide feedback to the coaching staff regarding player progress or fatigue. All players had passed the team’s mandatory pre-participation physical prior to the onset of the season. The players gave their informed consent as part of their sport requirements, which is consistent with the University of Central Florida’s policies for use of human subject research.

### Game Data Collection

Prior to each match, all athletes were fitted with a 10-Hz GPS device (Catapult, Minimax 4.3, Victoria, Australia) that was placed in a pocket of a vest worn under their game jersey during the match. The pocket was located between the athlete’s shoulder blades. Matches took place on a Friday at 7:00 PM local time (00:00 GMT) when in Orlando, FL and 5:00 PM local time (00.00 GMT) when in Colorado Springs, Colorado. The coaches followed their normal game routines, with no interventions performed to compensate for altitude change. Post-match data collected by the GPS device were downloaded for analysis using Catapult Sprint Software.

Analysis of the raw data files was performed post-match, and then saved to derive individual athlete reports. Each athlete’s physical activity was monitored on the field and defined across various thresholds (bands), with parameters set for each band (Labeled I–VII) (Table 2). Within each band, each athlete’s activity was measured by the time spent within that band width, the number of events at that threshold, and the percentage of the game that took place in each band. HIR was defined to include moderate and high speed running and sprinting (bands V through VII). Within the bands, the percentage of time, the rate of HIR and the rate of TDC during each half were examined.

### Statistical Analysis

Statistical analysis of the data was accomplished using a repeated measures analysis of variance (ANOVA). In the event of a significant F-ratio, LSD post-hoc tests were used for pairwise comparisons. A criterion alpha level of p ≤ 0.05 was used to determine statistical significance. Data were analyzed using SPSS v20 software (SPSS Inc., Chicago, IL). Prior to statistical procedures, all data were assessed for normal distribution, homogeneity of variance, and sphericity. All data are reported as mean ± SD.

## Results

**Figure 1:**
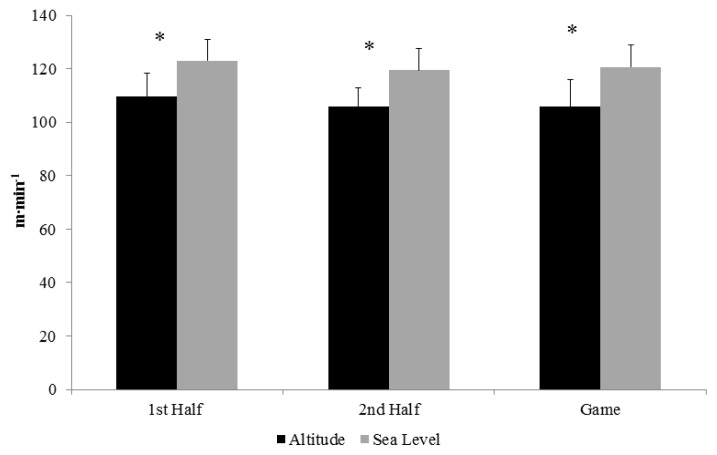
Total Distance Covered per Minute. * = Significant difference between altitude and sea level

Participants played significantly (p = 0.003) longer at sea level (83.24 ± 5.27 min) than at altitude (74.23 ± 2.93 min). The relative distance covered in a game (m·min^−1^ played) can be observed in Figure 1. The relative distance covered during the entire game at altitude was significantly less (p < 0.001) than that performed at sea level. Differences in distance covered per minute were also noted in the first (p = 0.006) and second (p = 0.003) halves.

**Figure 2:**
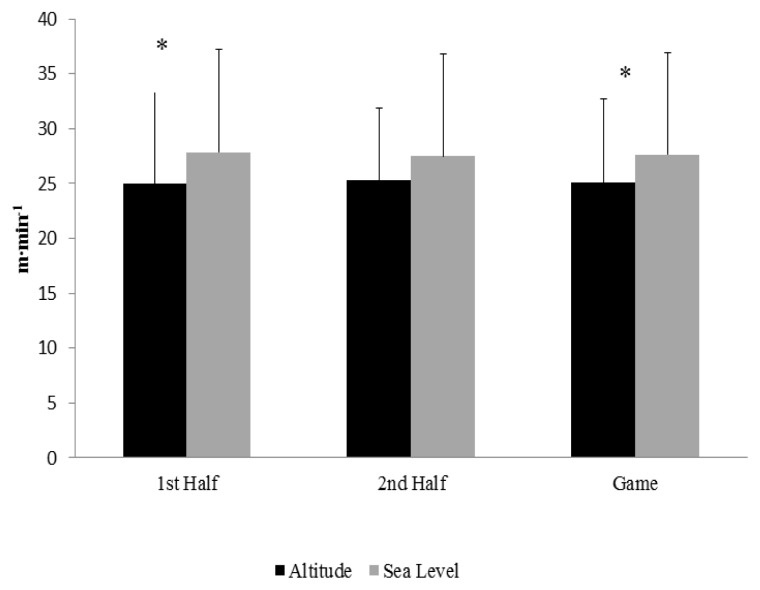
High Intensity Distance Covered per Minute. * = Significant difference between altitude and sea level

The relative distances performed at a high intensity threshold can be observed in Figure 2. During the first half the distance covered across these high intensity runs was significantly lower (p = 0.022) at altitude than at sea level. Although no differences (p = 0.288) were noted in the second half of the contests, the distance covered during high intensity runs for the entire game was significantly (p = 0.037) less at the moderate altitude than when playing at sea level. When examining the percent time spent at high intensity running, a significant difference (p = 0.039) was seen in the first half between the games played at a moderate altitude (9.0 ± 2.2%) compared to sea level (10.3 ± 3.2%). No difference was seen (p = 0.23) in the second half between the game played at a moderate altitude (9.3 ± 2.2%) compared to sea level (10.3 ± 3.1%). However, when comparing the percent time spent at high intensity runs for the entire game, the percentage of activity performed at a high intensity (10.4 ± 3.3%) during the matches performed at sea level tended to be greater (p = 0.064) than the percentage of high intensity activity performed at a moderate altitude (9.1 ± 2.2%).

## Discussion

The main findings of this study suggest that activity profiles of soccer players who reside at sea level and travel to a match at a moderate altitude (1800 m) are reduced. This is based upon differences observed in the total distance covered, and the distance covered during high intensity runs per minute between soccer games performed at altitude compared to the average performance seen during games at sea level. These effects appear to be consistent in both the first and second halves of the match. Although this appears to be the first investigation to compare activity profiles in contests occurring at both sea level and at a moderate altitude, it does support previous research results suggesting that teams residing at sea level are at a competitive disadvantage when they play at an altitude (McSharry, 2007). Teams native to altitude and playing at altitude are reported to have nearly a 30% greater probability of winning home games against opponents residing at sea level (McSharry, 2007). Although FIFA considered a ban on matches performed over 2,500 m, they only suggested that teams better prepare for games played at altitude through appropriate acclimatization (Bartsch et al., 2008). However, for intercollegiate matches the opportunity for athletes to acclimatize to altitude is very limited considering that multiple games are played weekly.

Previous studies examining high intensity exercise performance at altitude are very limited. Niess and colleagues (2003) reported that the acute stress response during high intensity interval training in endurance athletes was more pronounced at a moderate altitude (1800 m) than at sea level. Friedman and colleagues (2007) suggested that anaerobic capacity was not significantly changed during an acute exposure to a moderate altitude in endurance-trained athletes. However, a decrease in time to exhaustion during a maximal effort bout of exercise was attributed to the decrement in aerobic capacity. An examination on triathletes performing at various altitudes showed that high intensity performance (5-min time trials following a bout of interval exercise) was impaired at altitudes ranging between 2000 – 3200 m (Clark et al., 2007). In contrast, others have investigated the impact of a moderate altitude (2320 m) on repeat 400 m sprint performance with different rest intervals (1 min, 2 min, 5 min) and found no significant differences in accumulated oxygen deficit across the trials, suggesting that exercise at a moderate altitude did not inhibit anaerobic metabolism (Feriche et al., 2007). Although the majority of these studies suggest a greater risk for performance decrements and fatigue during exercise at a moderate altitude, none of these studies examined a protocol that simulated actual sport performance.

One study attempted to simulate rugby performance at 1550 m (Hamlin et al., 2008). They reported significant decrements (~16%) in repetitive explosive power and 20-m shuttle performance (~3%) at altitude compared to sea level. Another study examined male team sport athletes performing three sets of repeated sprints on a non-motorized treadmill at simulated altitudes (2000 m, 3000 m and 4000 m) and reported significant decreases in mean power output and significant increases in lactate concentrations at all altitudes compared to sea level (Goods et al., 2014). Our findings provide additional support to these investigations indicating the deleterious effects of performing at a moderate altitude. The present study though appears to be the first to examine actual game performance.

The significant declines in total distance covered and in distance of high intensity runs during a soccer game at a moderate altitude may be attributed to both a decrease in aerobic capacity and possibly a decline in anaerobic power and capacity in these athletes. In studies examining soccer athletes, both aerobic power and high intensity running performance were demonstrated to be predictive of game performance, and associated with the level of play (Bradley et al., 2009; Krustrup et al., 2005; Mohr et al., 2003). In a study examining NCAA Division I female soccer players, high intensity runs were associated with a high level of aerobic fitness (McCormack et al., 2014). That investigation also indicated that power output in female soccer players was also associated with high intensity running speed and frequency of high intensity exertions on the field, indicating the importance of anaerobic power performance in soccer. In addition, sustained high intensity running performance was also associated with VO2max (McCormack et al., 2014), demonstrating the need for substantial aerobic capacity during a sustained soccer match. Although aerobic capacity has a limited role in anaerobic activity, it may play a critical role in recovery following bouts of high intensity running (Hoffman, 1997). The decrease in total distance covered and the distance of high intensity runs observed during the game studied was likely a function of an impaired cardiovascular function associated with the moderate altitude.

The game played at the moderate altitude was also conducted at temperatures that were lower than the games played at sea level. However, the temperature readings during the game played at the moderate altitude have not been reported to present any significant performance limitations (Hoffman, 2014). Further, exercise performed in cold conditions (e.g. 8° – 10°C), similar to that seen in this study, have been shown to be less exhausting than exercise performed in the heat (Galloway and Maughan, 1997). Thus, performance differences observed in this study resulted from the sojourn to the moderate altitude, and less likely the result of any difference in ambient temperature.

This study provided a unique opportunity to examine the effect of a moderate altitude on athletes who reside at sea level during a competitive athletic event. Considering that only six athletes satisfied the inclusion criteria of minutes played per game, the results of this case study provide only a limited insight into the performance effects of non-acclimated athletes playing at a moderate altitude. The sample size was too small to provide any meaningful information whether altitude affects one position on the field more than the other. Considering differences in playing responsibility, this is an area of future exploration. A field study provides an interesting examination of athletes that take into account the competitive factors that cannot be duplicated in a laboratory setting. However, a laboratory study will provide greater control of minutes played, physiological limitations and enhance the sensitivity of performance measures.

The results of this study indicate that non-acclimatized soccer players performing at a moderate altitude may experience declines in total distance run, and their ability to sustain higher rates of running for the duration of the game. This may result in a competitive disadvantage for athletes that reside at sea level and travel to play at a moderate altitude. Future research should consider examining potential countermeasures to minimize this competitive disadvantage when acclimation is not possible.

## Figures and Tables

**Table 1:** Environmental Conditions at Examined Competitions

	Altitude (m)	Time (EDT)	Temperature (°C)	Conditions	Humidity	Wind Speed (km·h^−1^)
**Sea Level**	25	6:53 PM	27.2	Mostly Cloudy	72%	14.8
		7:53 PM	26.7	Clear	79%	13.0
		8:53 PM	26.1	Overcast	79%	11.1
**Sea Level**	25	6:53 PM	25.0	Clear	94%	Calm
		7:53 PM	25.6	Clear	90%	Calm
		8:53 PM	25.6	Clear	87%	5.6
**Altitude**	1839	6:54 PM	8.9	Scattered Clouds	83%	7.4
		7:54 PM	8.9	Scattered Clouds	87%	Calm
		8:54 PM	7.8	Scattered Clouds	86%	9.3

**Table 2:** Velocity Zones

Classification	Band	Low (m/s)	High (m/s)	Label
Rest/Recovery	I	0	0.56	Standing
	II	0.56	1.94	Walking
	III	1.94	2.5	Jogging
	IV	2.5	3.61	Low Intensity Running
High Intensity Running	V	3.61	4.44	Moderate Intensity Running
	VI	4.44	6.11	High Intensity Running
	VII	6.11	+	Sprinting
